# The Health Effects of Vitamin D and Probiotic Co-Supplementation: A Systematic Review of Randomized Controlled Trials

**DOI:** 10.3390/nu13010111

**Published:** 2020-12-30

**Authors:** Myriam Abboud, Rana Rizk, Fatme AlAnouti, Dimitrios Papandreou, Suzan Haidar, Nadine Mahboub

**Affiliations:** 1 Department of Health, College of Natural and Health Sciences, Zayed University, Dubai 19282, UAE; fatme.alanouti@zu.ac.ae (F.A.); dimitrios.papandreou@zu.ac.ae (D.P.); 2 Institut National de Santé Publique, d’Épidémiologie Clinique et de Toxicologie (INSPECT-Lb), Beirut, Lebanon; rana.rizk@inspect-lb.org; 3 Department of Nutrition and Food Sciences, Faculty of Arts and Sciences, Lebanese International University, Beirut 657314, Lebanon; suzan.haidar@liu.edu.lb (S.H.); nadine.baltagi@liu.edu.lb (N.M.); 4 Department of Health Promotion, Faculty of Health, Medicine and Life Sciences, Maastricht University, 6229 GT Maastricht, The Netherlands

**Keywords:** vitamin D, probiotic, supplementation, adults, randomized controlled trial, systematic review

## Abstract

Evidence of synergic health effects of co-supplementation with vitamin D and probiotics is emerging. Following the Preferred Reporting Items for Systematic Reviews and Meta-Analyses PRISMA statement, scientific databases and the grey literature were searched, and a narrative review and risk of bias assessment were conducted. Seven randomized controlled trials were included, which had low risk of bias. Six studies were double-blind, and once single-blind, extended over 6–12 weeks, and included 50–105 participants. Conditions explored included schizophrenia, gestational diabetes, type 2 diabetes and coronary heart disease, polycystic ovarian syndrome, osteopenia, irritable bowel syndrome (IBS), and infantile colic. Supplementation frequency was daily or bi-monthly, with mainly vitamin D3, and *Lactobacillus*, *Bifidobacterium*, and *Streptococcus*. Comparators were placebo, vitamin D, lower vitamin D dose, and probiotics and lower vitamin D dose. The co-supplementation yielded greater health benefits than its comparators did in all studies except in one assessing IBS. Beneficial effects included decreased disease severity, improved mental health, metabolic parameters, mainly insulin sensitivity, dyslipidemia, inflammation, and antioxidative capacity, and lower use of healthcare. Co-supplementation of vitamin D and probiotics generated greater health benefits than its comparators did. More studies in other diseases and various populations are needed to confirm these findings and to elucidate the optimal form, composition, and frequency of this co-supplementation.

## 1. Introduction

The gut microbiota refers to the assemblage of microorganisms, including bacteria, viruses, and fungi, located in the gastrointestinal (GI) tract [1]. There has been increasing emphasis on the role of the microbiota in physiology, suggesting that it can be considered as another human organ [2]. Furthermore, emerging evidence suggests that this invisible organ is a key driver of human health and disease. Gut microbiota plays a critical role in maintaining metabolic and immune health, synthesis of vitamins, obtaining inaccessible nutrients from the diet, renewal of epithelial cells, fat storage, maintaining intestinal barrier integrity, and brain development [3,4]. Dysbiosis, or alteration in the gut microbiota composition, is a crucial risk factor for the development of several disorders such as inflammatory bowel disease, obesity, diabetes, asthma, and allergies [5,6]. The gut microbiota composition is affected by intrinsic and extrinsic factors like genetics, age, dietary changes, in addition to physiological and psychological stress [2,7].

Specifically, vitamin D and the vitamin D receptor (VDR) were shown to modulate the gut microbiota [8]. Increased VDR expression may decrease microbial dysbiosis, enhance barrier function, increase the expression of antimicrobial peptides, decrease pro-inflammatory cytokines, and increase the commensal production of short-chain fatty acids [2,8]. Likewise, probiotics, which are ingestible nonpathogenic living microorganisms, were also shown to improve the balance of intestinal microbiota by regulating microbial components and metabolites [9]. Probiotics simulate the immune system, balance commensal and pathogenic bacteria, and reestablish homeostasis. They protect barrier integrity, alter toxic compounds, and host products. Thus, they ameliorate inflammation and prevent and repair cell damage [9].

Vitamin D deficiency and defects in VDR signaling have been related to several metabolic, cardiovascular, neurodevelopmental and cancer diseases [10,11]. Yet, interventional studies have conflicting evidence on the effect of vitamin D supplementation in their treatment [12,13,14,15,16]. Similarly, human probiotic supplementation studies generated conflicting evidence regarding the effectiveness of probiotics in the treatment of several health conditions such as allergies, GI disorders, metabolic syndrome, and obesity [17,18,19,20].

Recently, a promising evidence of synergic effects of combined supplementation with vitamin D and probiotics in modulating the gut microbiota and metabolome, in addition to fostering healthy microbe–host interactions, is emerging [9,21,22]. This co-supplementation holds a preventive and therapeutic potential with crucial clinical implications. Biologically plausible mechanisms support this interplay. Probiotics were shown to increase vitamin D intestinal absorption, and increase VDR protein expression and transcriptional activity [9]. Likewise, VDR status seems to be crucial in regulating the mechanisms of action of probiotics and modulating their anti-inflammatory, immunomodulatory and anti-infective benefits, suggesting a two-sided pathway [6,8].

The aim of this systematic review is to investigate the literature and summarize the available evidence of randomized controlled trials (RCTs) on the various health effects of a combined supplementation of vitamin D and probiotics among children and adults.

## 2. Materials and Methods

### 2.1. Review Design

The reporting of this systematic review followed the Preferred Reporting Items for Systematic Reviews and Meta-Analyses (PRISMA) statement [23]. A predefined protocol for this systematic review was registered at the OSF registries.

### 2.2. Criteria for Study Inclusion

Randomized controlled trials (RCTs) conducted on adults or children, healthy or with disease other than those known to influence vitamin D metabolism, and including an intervention group that received a co-supplementation of vitamin D and probiotics, and a control group of placebo, or a lower dose of vitamin D or probiotics, or a different form of vitamin D, or different strains of probiotics, were included in this systematic review. RCTs with a duration of a minimum of 1 month were included; this duration was deemed sufficient for the intervention to produce an effect. Additionally, RCTs involving other co-interventions were included, only if both arms received the same co-intervention.

Studies were excluded if they were non-randomized, uncontrolled, involving participants taking medication known to influence vitamin D metabolism or with conditions affecting vitamin D metabolism such as chronic kidney disease, chronic liver disease, or malabsorption states, or entailing a supplementation with either vitamin D or probiotics.

### 2.3. Search Strategy

The systematic search included Medical Subject Headings (MeSH) and keywords for three concepts: (1) vitamin D, (2) probiotics, and (3) randomized controlled trial, and was conducted in PubMed, MEDLINE, CINAHL, EMBASE, the Cochrane Library, ClinicalTrials.gov, and the International Clinical Trials Registry Platform (ICTRP), from inception until 4 November 2020, without language restrictions. The electronic search strategy, detailed in the Tables S1 and S2, was validated by a medical information specialist. Reference lists of included RCTs and relevant reviews were also hand-searched for eligible studies.

### 2.4. Study Selection

The titles and/or abstracts retrieved by the search were screened by two pairs of authors, and the full text of all relevant papers was assessed for eligibility independently and in duplicate. A calibration exercise was conducted before study selection to ensure the validity of the process. Inconsistencies were discussed amongst reviewers, and unresolved discrepancies were settled by a third reviewer.

### 2.5. Data Extraction

Data from the selected articles were extracted by two pairs of authors using a data extraction form. Changes from baseline for the intervention were compared with the control in all the parameters analyzed. A calibration exercise was first conducted. Disagreements were resolved through discussion or with the help of a third reviewer.

### 2.6. Quality Assessment

The risk of bias for the included studies was assessed using the Cochrane criteria (sequence generation, allocation concealment, blinding of participants and outcome assessors, incomplete outcome data, and selective outcome reporting) [24], whereby each potential source of bias was graded as low, high, or unclear risk. The process was carried out by two pairs of authors independently and in duplicate. They underwent a calibration exercise before performing the assessment of risk of bias. Conflicts were resolved through discussion amongst the pair of reviewers or through consultation with a third reviewer.

### 2.7. Data Synthesis

A narrative review of the findings was performed and is included in Table S2.

## 3. Results

### 3.1. Search Results

**Figure 1:**
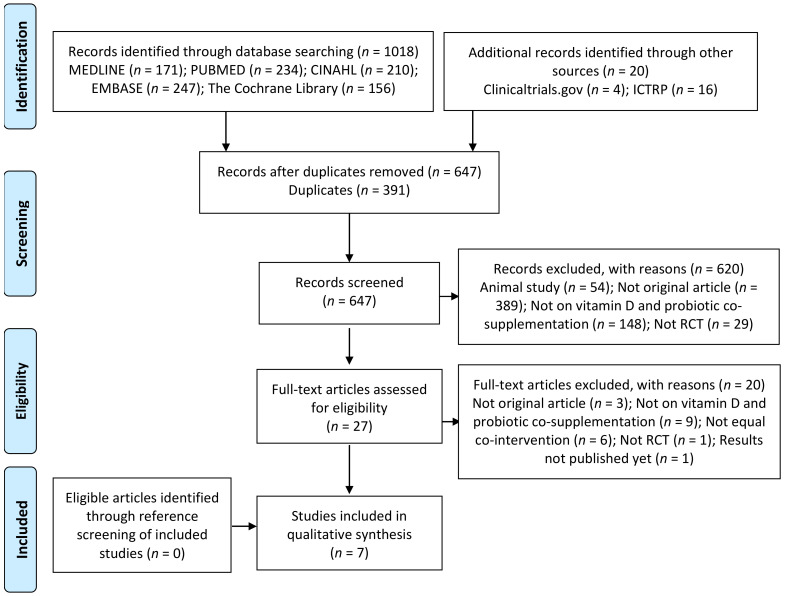
Preferred Reporting Items for Systematic Reviews and Meta-Analyses (PRISMA) Diagram of Study Selection.

Study selection process is detailed in Figure 1, whereby seven studies meeting the inclusion criteria were included in the systematic review.

### 3.2. Characteristics of Included Studies

Characteristics of included RCTs are detailed in Table 1. The studies were published between 2015 [25,26] and 2019 [27,28]. Five studies were conducted in Iran [27,28,29,30,31], one in Italy [25], and one in the United Kingdom [26]. All the studies were randomized double-blind [26,27,28,29,30,31], except for Savino et al. [25], which was single-blind. The duration of the studies ranged between 6 [29,31] to 12 weeks [25,26,27,28,30]. The number of participants ranged between 50 [31] and 105 [25]. The studies were conducted on infants [25], pregnant women [29], and other adults with diseases [26,27,28,30,31]. Health conditions that were studied included schizophrenia [27], gestational diabetes mellitus (GDM) [29], type 2 diabetes mellitus (T2DM) and coronary heart disease (CHD) [30], polycystic ovarian syndrome (PCOS) [28], osteopenia [31], irritable bowel syndrome [26], and infantile colic [25].

In the studies by Ghaderi et al. [27], Ostadmohammadi et al. [28], Raygan et al. [30] interventions consisted of a co-supplementation with vitamin D and probiotics, and the control group received placebo only [27,28,30]. In the study by Tazzyman et al. [26], the intervention group received a co-supplementation with vitamin D and probiotics, one of the control groups received a placebo, and the other one received placebo and vitamin D [26]. In Savino et al. [25], the intervention group received vitamin D and probiotics, but the control group received vitamin D only. In the study by Jafarnejad et al. [31], the intervention group received probiotics, yet vitamin D was supplemented in all groups. This co-intervention rendered the comparison between the intervention group receiving probiotics and vitamin D and the control group receiving placebo and a similar dose of vitamin D. Additionally, in the study by Jamilian et al. [29] the intervention consisted of a co-supplementation with vitamin D and probiotics; one of the control groups received probiotics, and the other one received placebo. Yet, in this study [29], all the groups also received a lower dose of vitamin D. This co-intervention rendered the comparison between the intervention group receiving probiotics and a high dose of vitamin D, the first control group receiving probiotics and a lower dose of vitamin D, and the second control group receiving placebo and a lower dose of vitamin D [29].

**Table 1:** Characteristics of included studies.

First Author, Year, Country	Study Design	Duration	Study Population	Intervention	Control	Co-Intervention	Compliance/Drop-out
Ghaderi, 2019, Iran [27]	Randomized, double-blind, placebo-controlled trial	12 weeks	*n* = 60, aged 25–65, 93.33% men, diagnosed with schizophrenia using DSM-IV-TR criteria with disease duration ≥2 years, PANSS score ≥55, treated with chlorpromazine (300–1000 mg/day, except clozapine) and anticholinergic agents (Trihexyphenidyl, 4–8 mg/day) during the last 6 months	Vitamin D3 and probiotic supplement: - Vitamin D3: 50,000 IU every 2 weeks; DDE = 3571.4 IU - Probiotics: 8 × 10^9^ CFU/day containing *Lactobacillus acidophilus, Bifidobacterium bifidum, Lactobacillus reuteri*, and *Lactobacillus fermentum* (each 2 × 10^9^ CFU/day)	Placebo similar shape and packaging	None	Compliance: >90%Drop out: I: 13.33% C: 13.33% (Intention-to-treat analysis)
Jafarnejad, 2017, Iran [31]	Randomized, double-blind, placebo-controlled clinical trial	6 weeks	*n* = 50, age 50–72 years, women with mild bone loss (osteopenia) diagnosed based on the World Health Organization criteria (T-score between −1.0 and −2.5)	Probiotic supplement: *Lactobacillus casei* 1.3 × 10^10^ CFU, *Bifidobacterium longum* 5 × 10^10^ CFU, *Lactobacillus acidophilus* 1.5 × 10^10^ CFU, *Lactobacillus rhamnosus* 3.5 × 10^9^ CFU, *Lactobacillus bulgaricus* 2.5 × 10^8^ CFU, *Bifidobacterium breve* 1 × 10^10^ CFU, and *Streptococcus thermophilus* 1.5 × 10^8^ CFU/500 mg	Placebo similar in shape, size, odor, color and packaging	Vitamin D (200 IU daily) and Calcium (500 mg daily)	Compliance 100% Drop out: I: 20% C: 16%
Jamilian, 2018, Iran [29]	Randomized, double-blind, placebo-controlled clinical trial	6 weeks	*n* = 87, women with GDM diagnosed by a “one-step” 2-h 75-g oral glucose tolerance test based on the ADA guidelines	Vitamin D and probiotic supplement: - Vitamin D: 50,000 IU every 2 weeks; DDE= 3571.4 IU - Probiotics: 8 × 10^9^ CFU/g probiotic containing *Lactobacillus acidophilus, Bifidobacterium bifidum, L. reuteri*, and *Lactobacillus fermentum* (each 2 × 10^9^ CFU/g)	C1: 8 × 10^9^ CFU/day of probiotic supplements C2: Placebo Similar in appearance, color, shape, size, odor, taste and packaging	Vitamin D3: 1000 IU and Vitamin B9: 400 mg, daily from the beginning of pregnancy, and Ferrous sulfate: 60 mg, daily from the secondtrimester	Compliance: 100% Drop out: I: 0% C1: 6.66% C2: 10%
Ostadmohammadi, 2019, Iran [28]	Randomized, double-blind, placebo-controlled clinical trial	12 weeks	*n* = 60, aged 18–40 years, women with PCOS, diagnosed based on the Rotterdam criteria with BMI: 17–34 kg/m^2^ and insulin resistance: 1.4–4	Vitamin D and probiotic supplement: - Vitamin D: 50,000 IU every 2 weeks; DDE = 3571.4 IU - Probiotics: 8 × 10^9^ CFU/day containing *Lactobacillus acidophilus, Bifidobacterium bifidum, Lactobacillus reuteri* and *Lactobacillus fermentum* (each 2 × 10^9^ CFU/g)	Placebo similar in appearance, color, shape, size, odor, taste and packaging	None	Compliance 100%; No drop out
Raygan, 2018, Iran [30]	Randomized, double-blind, placebo-controlled clinical trial	12 weeks	*n* = 60, age 45–85 years, 50% men, with T2DM diagnosed based on the criteria of the ADA and with CHD diagnosed as per the AHA with 2- and 3-vessel CHD	Vitamin D3 and probiotic supplement: - Vitamin D3: 50,000 IU every 2 weeks; DDE = 3571.4 IU - Probiotics: 8 × 10^9^ CFU/g - containing *Lactobacillus acidophilus, Bifidobacterium bifidum, Lactobacillus reuteri*, and *Lactobacillus fermentum* (each 2 × 10^9^ CFU/g)	Placebo similar in appearance, color, shape, size, odor, taste and packaging	None	Compliance > 90% Drop out: I: 13.33% C: 13.33% (Intention-to-treat analysis)
Savino, 2015, Italy [25]	Single-blind, randomized controlled, parallel-group trial	12 weeks	*n* = 105, newborns aged less than 10 days of life, 48.5% boys, with gestational age between 37 and 42 weeks, birth weight from 2500 to 4300 g, and normal physical examination	Vitamin D and probiotic supplement: - Vitamin D3: 400 IU daily - Probiotics: *Lactobacillus reuteri* DSM 17938 (10^8^ CFU)	Vitamin D (400 IU daily)	None	No infants lost to follow- ups
Tazzyman, 2015, United Kingdom [26]	Double-blind, randomized, three-arm parallel design trial	12 weeks	*n* = 51, 7.8% men, with previous clinical diagnosis of IBS and met the Rome III criteria and stratified according to vitamin D status at baseline (deficient: 25(OH)D <20 ng/mL; repleted: 25(OH)D >20 ng/mL)	Vitamin D3 and probiotic supplement: - Vitamin D3: sublingual liquid spray, 3000 IU daily - Probiotics: *Lactobacillus acidophilus*, CUL60 (NCIMB 30157), CUL21 (NCIMB 30156), *Bifidobacterium bifidum* CUL20 (NCIMB 30153) and *Bifidobacterium animalis subsp. lactis* CUL34 (NCIMB 30172) 2.5 × 10^10^ CFU per capsule	C1: Double placebo C2: Placebo and Vitamin D3 (400 IU daily) Similar in form, containing identical buffers	None	Compliance: 98% Drop out: 0%

25(OH)D: 25-hydroxyvitamin D; ADA: American Diabetes Association; AHA: American Heart Association; BMI: Body Mass Index; C: Control; CFU: Colony Forming Units; CHD: Coronary Heart Disease; DDE: Daily Dose Equivalent; DSM-IV-TR: Diagnostic and Statistical Manual of Mental Disorders, Fourth Edition, Text Revision; GDM: Gestational Diabetes Mellitus; I: Intervention; IBS: Irritable Bowel Syndrome; IU: International Unit; PANSS: The Positive and Negative Syndrome Scale; PCOS: Polycystic Ovary Syndrome; T2DM: Type 2 Diabetes Mellitus; TDD: Total Daily Dose.

The frequency of supplement administration ranged between daily [25,26,31] and bi-monthly [27,28,29,30]. Probiotic supplementation was given in the form of a capsule in all studies [25,26,27,28,29,30,31], whereas supplementation of vitamin D was either in the form of a capsule [26,27,28,29,30,31] or sublingual liquid spray [25]. The form of vitamin D supplemented was not specified in the studies by Jamilian et al. [29], Ostadmohammadi et al. [28], and Jafarnejad et al. [31], and studies by Ghaderi et al. [27], Raygan et al. [30], Tazzyman et al. [26], and Savino et al. [25] used vitamin D3, and the daily dose equivalent ranged from 200 International Units (IU) [31] to 4571.4 IU [29]. Probiotic strains that were investigated included *Lactobacillus* in all the studies [25,26,27,28,29,30,31], *Bifidobacterium* in all the studies [26,27,28,29,30,31] except for the one by Savino et al. [25], and *Streptococcus* only in Jafarnejad et al. [31]. The supplemented doses greatly varied across studies, and in the majority of the studies, it consisted of 8 × 10^9^ Colony Forming Units (CFU) per day.

There was a high rate of compliance in all studies [25,26,27,28,29,30,31], and the drop-out rate ranged from 0% [25,26,28,29] to 20% [31], and was almost equal between the compared groups in all studies [25,26,27,28,30,31], except in Jamilian et al. [29].

### 3.3. Assessment of Risk of Bias

Risk of bias assessment of included RCTs is available in Table 2. In general, the quality of the RCTs design and reporting was high. In all studies [25,26,27,28,29,30,31], random allocation of participants was adequate, and allocation was concealed. Blinding of participants and personnel was reported in all of the included studies [26,27,28,29,30,31], except in the one by Saviano et al. [25], where both patients and physicians, except outcome assessors, were aware of their allocation. All studies reported complete outcome data [25,26,28,29,31], except for the studies conducted by Ghaderi et al. [27] and Raygan et al. [30] who did not mention how missing data were dealt with. Finally, in all studies [25,26,27,28,29,30,31], all pre-specified outcomes were reported on.

### 3.4. Results of Included Studies

The outcomes assessed and the findings of included RCTs are presented in Table 3. In Ghaderi et al. [27], Ostadmohammadi et al. [28], Raygan et al. [30], and Savino et al. [25], co-supplementation with probiotics and vitamin D yielded greater health benefits than either placebo [27,28,30] or vitamin D on its own [25]. Specifically, in Ghaderi et al. [27], the co-supplementation, compared with placebo, had a favorable effect on schizophrenia symptoms severity, as well as other metabolic outcomes, mainly insulin sensitivity, inflammation, and antioxidative capacity. In Ostadmohammadi et al. [28], vitamin D and probiotic co-supplementation in women with PCOS, compared with placebo, had beneficial effects on mental health parameters, namely depression, anxiety and stress, as well as hormonal, inflammatory, and antioxidative parameters, and on the symptoms of PCOS, specifically, hirsutism. However, the co-supplementation was not associated with improvements in sex hormone-binding globulin, nor with other symptoms of PCOS, namely acne and alopecia, nor were there improvement in sleep quality [28]. In Raygan et al. [30], combined supplementation with vitamin D and probiotics for people with T2DM and CHD, compared with placebo, improved anxiety and depression, insulin sensitivity, inflammatory markers, antioxidative capacity and dyslipidemia, specifically high-density lipoprotein-cholesterol. However, this intervention did not result in a better control of fasting glucose, other markers of dyslipidemia, specifically triglycerides, very low and low lipoprotein-cholesterol, nor with blood pressures [30]. In the study by Savino et al. [25], compared with vitamin D supplementation alone, vitamin D and probiotic co-supplementation to newborns was associated with a reduction of more than two pediatric consultations and phone calls regarding infantile colic over a 12-week period. The co-supplementation was also associated with a lower use of pain-relieving agents and of infant formula [25].

In the study by Jamilian et al. [29], all women with GDM in all groups were being supplemented with 1000 IU (low dose) vitamin D. The group supplemented with probiotics and high dose vitamin D, compared with placebo and low dose vitamin D, showed greater improvement in glucose control, insulin sensitivity, dyslipidemia, inflammatory markers, and antioxidative capacity [29]. Additionally, upon birth, newborns of mothers in this arm had lower incidence of both hyperbilirubinemia and hospitalization [29]. Moreover, the group supplemented with probiotics and high dose vitamin D, compared with probiotics and low dose vitamin D, exhibited a greater improvement in dyslipidemia, inflammation and antioxidative capacity [29]. Furthermore, newborns had better health outcomes [29]. Similarly, in the study by Jafarnejad et al. [31], all groups received 200 IU of vitamin D, and the group receiving probiotics had improvement in osteopenia markers (bone resorption and turnover), namely, bone-specific alkaline phosphatase, collagen type 1 cross-linked C-telopeptide, tumor necrosis factor α, and parathyroid hormone, but did not show an improvement in bone mineral density nor other serum indicators of osteopenia [31], compared with the group receiving placebo and vitamin D.

The only study where the co-supplementation was not found to be more effective than its comparators was the one conducted by Tazzyman et al. [26], where no significant difference in the symptoms of irritable bowel syndrome (IBS) was evident, between co-supplementation with probiotics and vitamin D, compared with vitamin D alone, or with placebo.

## 4. Discussion

So far, probiotic or vitamin D trials have shown major inconsistency in preventive or therapeutic effects on various health outcomes. The emergence of promising experimental studies on the interplay between vitamin D/VDR and probiotics in modulating the gut microbiota and influencing health and disease has led to several clinical trials of a combined supplementation in human subjects. Our exhaustive search identified seven eligible studies, which were included in our review. Our results show that a combined supplementation with vitamin D and probiotics was mostly more beneficial than placebo, vitamin D or probiotics alone in improving health outcomes in various populations, and suggest a dose-dependent effect.

Vitamin D deficiency had long been seen as a concern in metabolic and inflammatory disorders [32,33,34]. In the included studies, the majority of inflammatory markers improved with the co-supplementation. It is now evident that VDR expression regulates responses to inflammation through numerous mechanisms, such as inhibiting the nuclear factor-kappa B (NF-ĸB) pathway and activating autophagy [6]. VDR has an essential role for innate immune cells in intestinal inflammation, whereby the deletion of VDR in macrophages and granulocytes significantly increases the expression of pro-inflammatory cytokines in the colon [35]. In contrast, VDR signaling stimulates anti-inflammatory cytokine secretion [36]. Being a transcription factor, VDR can regulate the expression and signaling of target genes involved in intestinal inflammation and dysbiosis, such as *Atg16l1* [6]. A genome-wide association study of the gut microbiota showed that VDR gene variation in humans influences the intestinal microbiota [37]. Genetic variation at the VDR locus significantly influences microbial co-metabolism and the gut–liver axis [37]. Another study in VDR knockout mice found that the lack of VDR in the intestine leads to dysbiosis, with profound alterations in the gut microbiome profile characterized by an increased abundance of *Bacteroidaceae* [38]. However, to date, the mechanisms behind the change of human VDR protein after using vitamin D supplementation and its role in regulating the gut microbiome in health and inflammation are not entirely known [6]. In parallel, the anti-inflammatory markers and properties of probiotics are reliant on VDR expression [39]. There are data showing that probiotic treatment enhances VDR expression and activity in the host. In a probiotic mono-associated pig model, treatment with *Lactobacillus plantarum* in cultured intestinal epithelial cells resulted in an increase in VDR expression and cathelicidin mRNA [39]. Other data show that probiotics did not inhibit inflammation in mice lacking VDR [39]. Future research is needed to enhance our understanding of the complex interplay of nuclear receptors and probiotics, specifically VDR’s contribution to probiotic-induced anti-inflammation and its potential role in inflammatory conditions such as inflammatory bowel diseases [39].

Besides, our review documented improvement in insulin sensitivity, anti-oxidative patterns, and dyslipidemia markers with co-supplementation of vitamin D and probiotics. The same positive direction was also highlighted elsewhere [6,8]. Previous research documented a functional link existing between probiotic metabolism and nuclear receptors involved in regulating insulin sensitivity [22]. In a mice model of genetic dyslipidemia and intestinal inflammation, supplementation with a mixture of probiotic strains, including *Streptococcus thermophiles*, *Bifidobacterium breve*, *Bifidobacterium lactis*, *Lactobacillus acidophilus*, *Lactobacillus plantarum*, *Lactobacillus paracasei*, and *Lactobacillus helveticus* modified the nuclear receptors’ expression including VDR, and caused their direct transactivation, leading to reversing insulin resistance in liver and fat tissues and protecting against steatohepatitis and atherosclerosis [40]. Yet, these results although emanating from high-quality studies, are far from being conclusive, and future trials are needed before we can confidently establish the effectiveness and superiority of this co-supplementation.

More human experimental studies are needed to fully elucidate the interplay between nuclear receptors and probiotics in metabolic diseases. Shaping our understanding of this unexplored path might pave the way for multi-target preventive and therapeutic strategies, especially in situations where dietary and lifestyle changes have failed [22].

Additionally, improvement in mental health has been reported in this review. Vitamin D is involved in numerous brain processes including neuroimmunomodulation, neuroprotection, as well as brain development; all of which suggests a link between vitamin D and mental health [41,42]. Vitamin D may positively affect mental health through up-regulating tyrosine hydroxylase gene expression and increasing bioavailability of key neurotransmitters, such as norepinephrine and dopamine [43]. In parallel, mechanisms through which gut bacteria can affect mental status include microflora biosynthesis and the regulation of neurotransmitters, including serotonin [44] and gamma aminobutyric acid (GABA) [45]. Existing evidence also pinpoint an association between mood disorders and gut microbiota, and specify a role of the gut–brain axis in the physiopathology of clinical depression [46]. It is highly plausible that the synergism in vitamin D and probiotics’ anti-inflammatory, antioxidant, and immunomodulatory effects might augment their impact on mental health. This is yet to be confirmed by future interventional human studies.

The only study in this review that reported null results with the co-supplementation was a trial by Tazzyman et al. [26] which did not show any improvement in the symptoms of patient with IBS whose vitamin D was repleted. This study had a limited sample size (underpowered trial), and a limited duration of follow-up. Additionally, in that study, the group receiving placebo showed an improvement in vitamin D levels, which might be due to seasonal differences in sun exposure, and a placebo effect was observed on symptom scores. The authors speculated that increased sunlight exposure had increased vitamin D levels which in turn improved IBS symptoms. All of these limitations may have prevented the authors from detecting a significant difference in symptom scores between the placebo and supplemented groups. Additionally, individuals might need higher doses of vitamin D plus probiotic supplementation for a longer period of time to provide appropriate circulating levels for improving symptoms.

Understanding the mechanisms of the interplay between vitamin D and probiotics in modulating the gut microbiota and regulating host responses, and exploring the effectiveness of this form of supplementation in high-quality human studies are crucial before applying it to prevent and manage disease. Studies included in this review had revealed thoroughly the superiority of co-supplementing with vitamin D and probiotics. Vitamin D has shown benefits in cellular restoration and reducing inflammation. The latter has been implicated in the pathophysiology of an unlimited number of conditions and diseases. VDR expression and transcriptional activity can be a research focus for future genetic studies. In parallel, data about probiotics and their role in optimizing microbiota and absorption pathways would be very useful not only for vitamin D but for many other nutrients or enzymes involved to boost immunity and host response.

## 5. Strengths and Limitations

To our knowledge, this is the first review to systematically compile human interventional evidence on the effectiveness of a combined supplementation of vitamin D and probiotics. Our review has numerous strengths [47]. It was conducted following standard methods for reporting systematic reviews [23], and according to a pre-defined protocol, which was published a priori. To increase the comprehensiveness of our search, we searched multiple scientific databases and the grey literature, and did not limit our search to any publication language or time. All the steps of study inclusion, data extraction and quality assessment were conducted in duplicate. We only included RCTs, and assessed their risk of bias using a validated tool; and, in general, the included studies were of high quality. However, included trials were limited in number, and conditions assessed. They were also limited by the small sample size, and short duration of follow-up. Moreover, only two studies [25,26] provided details regarding the strain of bacteria in the used probiotics. None of the studies provided analyses of the gut microbiota, disabling us from establishing whether the co-supplementation changed the composition of the microbiota, or ascertaining whether the observed changes were due to changes in the gut microbiota. Furthermore, we could not pool the studies in a meta-analysis due to the heterogeneity in the populations, conditions assessed, outcomes, doses and forms of vitamin D supplemented, and doses and strains of probiotics supplemented.

## 6. Conclusions

A combined supplementation with vitamin D and probiotics seems to play a role on the physiological and psychological attributes of the human body, and represents a novel insight in the management of chronic diseases. The findings of this systematic review suggest a superiority of vitamin D and probiotics supplementation over placebo, vitamin D or probiotics alone, and propose a dose-dependent effect. However, solid conclusions cannot be drawn at this level, and these findings remain certainly not robust enough and should be interpreted with caution. Future high-quality studies in other disease areas and various populations are needed to confirm these findings and to inform on the form, composition, and frequency of this co-supplementation for optimal outcomes.

## Figures and Tables

**Table 2:** Risk of bias of included studies from consensus between a pair of raters.

First Author, YEAR	Random Sequence Generation (Selection Bias)	Allocation Concealment (Selection Bias)	Blinding of Participants and Personnel (Performance Bias)	Blinding of Outcome Assessment (Detection Bias)	Incomplete Outcome Data (Attrition Bias)	Selective Reporting (Reporting Bias)	Other Bias
Ghaderi, 2019 [27]	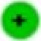	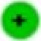	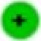	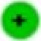	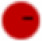	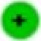	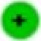
Jafarnejad, 2017 [31]	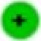	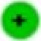	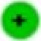	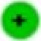	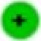	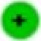	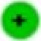
Jamilian, 2018 [29]	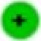	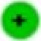	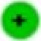	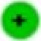	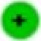	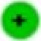	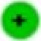
Ostadmohammadi, 2019 [28]	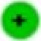	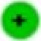	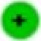	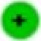	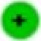	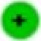	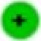
Raygan, 2018 [30]	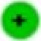	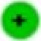	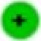	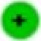	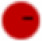	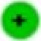	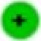
Savino, 2015 [25]	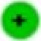	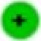	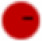	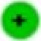	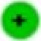	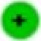	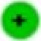
Tazzyman, 2015 [26]	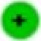	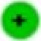	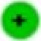	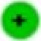	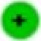	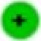	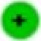

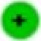
Low risk of bias 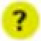
Unclear risk of bias 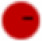
High risk of bias.

**Table 3:** Outcomes and results of included studies.

First Author, Year, Country	Outcome Measures	Results	Conclusion
Ghaderi, 2019, Iran ^1^ [27]	BMI: weight in kg divided by height in meters squared (height and weight measured withoutshoes and in light clothing by a trained staff) Serum 25-hydroxyvitamin D: ELISA kit Severity of psychiatric symptoms: PANSS Domains of cognitive function: BPRS scores TAC: method of ferric reduction antioxidant power developed by Benzie and Strain GSH: Beutler method MDA: Thiobarbituric acid reactive substances spectrophotometric Test Serum hs-CRP: ELISA kit NO: Griess Method Serum insulin: ELISA kit HOMA-IR and QUICKI: calculated using standard formula FPG and lipid profiles: Enzymatic kits	At baseline and end line: No significant difference between-groups, in height, age, weight, BMI and METs At baseline: Significant difference between-groups for positive PANSS score, BPRS, GSH and plasma NO At end line: In the I group compared with the C group: Significant greater decrease in MDA (−0.3 ± 0.9 vs. +0.2 ± 0.4 μmol/L), serum hs-CRP (−2.3 ± 3.0 vs. −0.3 ± 0.8 mg/L), FPG (−7.0 ± 9.9 vs. −0.2 ± 9.9 mg/dL), serum insulin (−2.7 ± 2.3 vs. +0.4 ± 2.0 μIU/mL), HOMA-IR (−0.8 ± 0.7 vs. + 0.1 ± 0.7), TG (−7.8 ± 25.2 vs. +10.1 ± 30.8 mg/dL), TC (−4.9 ± 15.0 vs. +5.9 ± 19.5 mg/dL), and TC/HDL-C (−0.1 ±0.6 vs. +0.3 ± 0.8) Significant greater increase in 25-hydroxyvitamin D (+9.1 ± 4.1 vs. +0.2 ± 0.4 ng/mL), general PANSS score (−3.1 ± 4.7 vs. +0.3 ± 3.9), total PANSS score (−7.4 ± 8.7 vs. −1.9 ± 7.5), plasma TAC (+51.1 ± 129.7 vs. −20.7 ± 53.3 mmol/L), QUICKI (+0.02 ± 0.01 vs. +0.0003 ± 0.01) No significant difference in the change of BPRS score and other metabolic profilesIn the analysis adjusting for baseline values of biochemical parameters, age and BMI, and controlling for potential confounders: The difference in changes in TC/HDL between the two groups became non-significant The difference in changes in negative PANSS score, BPRS and plasma GSH became statistically significant Other metabolic profiles did not change statically	Probiotic and vitamin D co-supplementation for 12 weeks to patients with chronic schizophrenia had beneficial effects on the general and total PANSS scores, as well as their metabolic profiles, compared with placebo
Jafarnejad, 2017, Iran [31]	Nutrient intake: 3-day dietary recall (2 weekdays and one weekend day), through monthly interview throughout the study period; nutrient analysis: by Nutritionist IV software modified for Iranian foods Physical activity: daily physical activity questionnaires validated by Kelishady et al. and calculated as metabolic equivalents/day Body weight: measured wearing light clothes without shoes using digital scales with 100-g precision Height: measured using a stadiometer with 0.5-cm precision in a normal standing position without shoes. BMI: weight in kilograms divided by height in meters squared BMD: dual energy X-ray absorptiometry Bone and pro-inflammatory biomarkers (TNF-α and IL-1b), Total serum levels of BALP, Osteocalcin, CTX, Vitamin D, RANKL, Osteoprotegrin, Serum TNF-α and IL-1b, Serum PTH, Urinary deoxypyridinoline: ELISA kits Serum calcium, phosphorus, magnesium, albumin, creatinine, alkaline phosphatase, and urinary amounts of calcium, phosphorus, magnesium, and creatinine: Pars Azmoon kits	At baseline: No significant differences between-groups At end line: Significant between-group differences in BALP (U/L) (I: 19.65 ± 1.66 at baseline and 16.53 ± 0.90 at end line vs. C: 17.81 ± 1.35 at baseline and 18.63 ± 1.29 at end line); CTX (ng/mL) (I: 0.41 ± 0.02 at baseline and 0.35 ± 0.02 at end line vs. C: 0.45 ± 0.02 at baseline and 0.42 ± 0.02 at end line); TNF-α (pg/mL) (I: 4.24 ± 0.5 at baseline and 3.73 ± 0.43 at end line vs. 3.83 ± 0.47 at baseline and 4.32 ± 0.5 at end line); PTH (pg/mL) (I: 31.92 ± 1.39 at baseline and 29.05 ± 1.53 at end line vs. C: 30.65 ± 1.44 at baseline and 32.81 ± 1.72 at end line) No significant between-group difference in Spinal BMD, Total hip BMD, RANKL, osteoprotegrin, RANKL/ osteoprotegrin ratio, deoxypyridinoline, osteocalcin, IL-1, Vitamin D, serum calcium, 24-h urinary Calcium, Serum phosphorus, 24-h urinary phosphorus, Serum magnesium, 24-h urinary magnesium, Serum creatinine, 24-h urinary creatinine, ALP, Albumin	Supplementation with probiotics, vitamin D and calcium for 6 weeks to postmenopausal osteopenic women showed a possible role in suppressing bone resorption and bone turnover, but did not affect bone density and other serum indicators compared with placebo, vitamin D and calcium
Jamilian, 2018, Iran ^2,3^ [29]	BMI: weight in kg divided by height in meters squared (height and weight measured withoutshoes and in light clothing by a trained staff) Polyhydramnios: sonographic estimation method at post-intervention and defined as an AFI in excess of 25 cm Preterm delivery: defined as delivery occurred at <37 weeks of pregnancy Newborn’s macrosomia: defined as birth weight of >4000 g. 2.5 Serum 25-hydroxyvitamin D: ELISA kit Serum insulin: ELISA kit HOMA-IR and QUICKI: calculated according to the standard formula FPG, serum TG, VLDL-C, TC, LDL-C and HDL-C: enzymatic kits Serum hs-CRP: ELISA kit Plasma NO: Griess method TAC: method of ferric reducing antioxidant power developed by Benzie and Strain GSH: Beutler method MDA: Thiobarbituric acid reactive substances spectrophotometric Test Newborns’ hyperbilirubinemia: when the total serum bilirubin levels were at ≥15 mg/dL (257 mmol/L) among infants 25–48 h old, 18 mg/dL (308 mmol/L) in infants 49–72 h old, and 20 mg/dL (342 mmol/L) in infants >72 h old	At baseline and end line: No significant difference between-groups, in age, height, weight, BMI, METs and intakes of macro- and micronutrients At end line: In the I group compared with the C1 group Significant greater decrease in TG (β −15.82 mg/dL), VLDL-C (β −3.16 mg/dL) and hs-CRP (β −0.32 mg/L) Significant greater increase in serum 25-hydroxyvitamin D (β 16.16 ng/mL), TAC (β 63.26 mmol/L) and GSH (β 53.61 mmol/L)Lower incidence of hyperbilirubinemiain newborns (10.0% vs. 13.8%) Lower incidence of newborns’ hospitalization (10.0% vs. 10.3%) No significant changes in other pregnancy outcomes In the I group compared with C2 group: Significant greater decrease in FPG (β −10.99 mg/dL), serum insulin (β −1.95 mIU/mL), HOMA-IR (β −0.76; 95%), TG (β −37.56 mg/dL), VLDL-C (β −7.51 mg/dL), HDL/TC B: −0.52), hs-CRP (β −1.80 mg/L) and MDA (β −0.43 mmol/L) Significant greater increase in 25-hydroxyvitamin D (β 18.21 ng/mL), QUICKI (β 0.01) HDL-C (β 4.09 mg/dL) and TAC (β 97.77 mmol/L) No significant changes in other metabolic parameters Lower incidence of hyperbilirubinemia in newborns (10.0% vs. 35.7%) Lower incidence of newborns’ hospitalization (10.0% vs. 32.1%) No significant changes in other pregnancy outcomes In the C1 group compared with the C2 group Significant greater decrease in FPG (β −8.60 mg/dL), Insulin (β −1.34 μIU/mL), HOMA-IR (β −0.54), TG (β −21.73 mg/dL), VLDL-C (β −4.34 mg/dL) and hs-CRP (β −1.36 mg/L), and MDA (β −0.50 μmol/L) Significant greater increase in serum 25-hydroxyvitamin D (β 2.05 ng/mL)	High dose of vitamin D and probiotic co-supplementation for 6 weeks to women with GDM had beneficial effects on metabolic status and newborns’ outcomes compared with placebo and low dose of vitamin D or probiotic supplementation and a low dose of vitamin D
Ostadmohammadi, 2019, Iran 2,3 [28]	Hirsutism: mFG scoring system Mental health: BDI, GHQ-28 and DASS Quality of sleep: PSQI Serum 25-hydroxyvitamin D: ELISA kit Serum total testosterone and SHBG: ELISA kits hs-CRP: ELISA kit Plasma NO: Griess method TAC: Benzie and Strain method GSH: Beutler method MDA: Thiobarbituric acid reactive substances spectrophotometric Test	At baseline: No significant difference between-groups for mean age, height and dietary macro- and micro-nutrient intakes. At end line: In the I group compared with the C group: Significant greater decrease in BDI (β −0.58), GHQ (β − 0.93), DASS (β − 0.90), total testosterone (β − 0.19 ng/mL), hirsutism (β − 0.95), hs-CRP (β − 0.67 mg/L) and MDA (β − 0.25 μmol/L) Significant greater increase in TAC (β 82.81 mmol/L) and GSH (β 40.42 μmol/L) No significant effect on serum SHBG and plasma NO levels, acne, alopecia and PSQI	Vitamin D and probiotic co-supplementation for 12 weeks to women with PCOS had beneficial effects on mental health parameters, but did not affect serum SHBG, plasma NO levels, acne, alopecia and PSQI, compared with placebo
Raygan, 2018, Iran 1 [30]	Serum 25-hydroxyvitamin D: ELISA FPG and lipid profiles: Enzymatic kit Insulin: ELISA kit HOMA-IR and QUICKI: standard formula Hs-CRP: ELISA kit Plasma TAC: Benzie and Strain method GSH: Beutler and Gelbart method MDA: spectrophotometric test NO: Griess method SBP and DBP: sphygmomanometer (Not detailed) Mental health: BDI, BAI, GHQ-28	At baseline and end line: No significant differences between-groups in mean age, height, weight, BMI and METs and macro and micronutrient intakes At end line: In the I group compared with the C group: Significant greater decrease in BDI (−2.8 ± 3.8 vs. −0.9 ± 2.1), BAI (−2.1 ± 2.3 vs. −0.8 ± 1.4) and GHQ scores (−3.9 ± 4.1 vs. −1.1 ± 3.4), Insulin (μIU/mL) (−2.8 ± 3.8 vs. +0.2 ± 4.9), HOMA-IR (−1.0 ± 1.6 vs. −0.1 ± 1.5), and hs-CRP (ng/mL) (−950.0 ± 1811.2 vs. +260.5 ± 2298.2) Significant greater increase in 25-hydroxyvitamin D (ng/mL) (+11.8 ± 5.9 vs. +0.1 ± 1.4), QUICKI (+0.03 ± 0.04 vs. −0.001 ± 0.01), serum HDL-cholesterol (mg/dL) (+2.3 ± 3.5 vs. −0.5 ± 3.8), plasma NO (μmol/L) (+1.7 ± 4.0 vs. −1.4 ± 6.7) and plasma TAC (mmol/L) (+12.6 ± 41.6 vs. −116.9 ± 324.2) No significant different changes in FPG, Triglycerides, VLDL-Cholesterol, LDL-Cholesterol, GSH, MDA, SBP and DBP	Vitamin D and probiotic co-supplementation for 12 weeks to diabetic people with CHD had beneficial effects on mental health, glycemic control, HDL-cholesterol levels, hs-CRP, NO and TAC, but did not affect other metabolic profiles and blood pressures, compared with placebo
Savino, 2015, Italy [25]	Administration of pain-relieving agents (cimetropium bromide at least three times per week or simethicone at least five times per week): daily reporting by parents % of infants switching from exclusive breastfeeding to partial or exclusive formula feeding: not detailed Number of phone-calls and visits due to infantile colic: noted by the pediatrician.	In the I group compared with the C group: - Significantly lower use of pain-relieving agents: Cimetropium bromide: RR: 0.04 (95%CI: 0.01–0.31); Simethicone: RR: 0.24 (95%CI: 0.14–0.41) - Significantly lower use of infant formula: RR: 0.37 (95%CI: 0.17–0.80) - Significantly lower number of calls to the pediatrician: 5.04 ± 2.64 vs. 8.40 ± 3.58 - Significantly lower number of visits in the pediatric ambulatory: 2.66 ± 1.77 vs. 4.98 ± 1.89	Vitamin D and probiotic co-supplementation for 12 weeks to newborns was associated with a reduction of pediatric consultations for infantile colic, use of pain-relieving agents and of infant formula, compared with vitamin D supplementation
Tazzyman, 2015, United Kingdom [26]	Serum 25(OH)D: Cobas e411 automated immunoassay Dietary intake: Food frequency questionnaire analyzed using FETA open source software IBS symptom: questionnaire assessing abdominal pain (pain severity and number of days with pain), bloating, bowel habits (minimum and maximum bowel movement per day and satisfaction with bowel habit) and quality of life	At baseline: No significant differences between-groups At end line: In the I and C2 groups compared with the C1 group: - Significantly higher 25OHD (ng/mL) (37.2 ±9.3 and 37.1 ± 11.7 vs. 25.3 ± 8.0) No significant between-group differences for any symptom tested, and total symptom severity (same results obtained for participants who were 25(OH)D-deficient at baseline)	Vitamin D and probiotic co-supplementation had no significant effect on the symptoms of IBS, compared with vitamin D alone, or placebo

25(OH)D: 25-hydroxyvitamin D; AFI: Amniotic Fluid Index; BAI: Beck Anxiety Inventory; BALP: Bone-Specific Alkaline Phosphatase; BDI: Beck Depression Inventory; BMD: Bone Mineral Density; BMI: Body Mass Index; BPRS: Brief Psychiatric Rating Scale; C: Control; CHD: Coronary Heart Disease; CI: Confidence Interval; CXT: Collagen Type 1 Cross-Linked C-Telopeptide; DASS: Depression Anxiety and Stress Scale; DBP: Diastolic Blood Pressure; ELISA: Enzyme-Linked Immunosorbent Assay; FBG: Fasting plasma glucose; GDM: Gestational Diabetes Mellitus; GHQ-28: General Health Questionnaire-28; GSH: Total Glutathione; HDL-C: High-Density Lipoprotein Cholesterol; HOMA-IR: Homeostasis Model of Assessment-Insulin Resistance; hs-CRP: High-Sensitivity C-Reactive Protein; I: Intervention; IBS: Irritable Bowel Syndrome; IL: Interleukin; LDL-C: Low-Density Lipoprotein Cholesterol; MDA: Malondialdehyde; MET: Metabolic Equivalent; mFG: modified Ferriman-Gallwey; NO: Nitric oxide; PCOS: Polycystic Ovary Syndrome; PSQI: Pittsburgh Sleep Quality Index; PTH; Parathyroid Hormone; QUICKI: Quantitative Insulin Sensitivity Check Index; RANKL: Serum Total Receptor Activator of Nuclear Factor-kB Ligand; RR: Relative Risk; SBP: Systolic Blood Pressure; SHGB: Sex Hormone-Binding Globulin; T2DM: Type 2 Diabetes Mellitus; TAC: Total Antioxidant Capacity; TC: Total cholesterol; TG: TNF: Tumor Necrosis Factor; Triglycerides; VLDL-C: Very Low-Density Lipoprotein Cholesterol. ^1^ Significance obtained for the time × group interaction, computed by analysis of the one-way repeated measures ANOVA. ^2^ Outcome measures refer to the change in values of measures of interest between baseline and end line in each group. ^3^ β: difference in the mean outcomes measures between treatment groups, and significance obtained from multiple regression model (adjusted for baseline values of each variable).

## Supplementary Materials

The following are available online at https://www.mdpi.com/2072-6643/13/1/111/s1, Table S1: Characteristics of included studies, Table S2: Outcomes and results of included studies.
