# Supplementary material for: The Health Effects of Vitamin D and Probiotic Co-Supplementation: A Systematic Review of Randomized Controlled Trials
Source: Nutrients. 2020 Dec 30;13(1):111. doi: 10.3390/nu13010111 (PMC7824176; doi:10.3390/nu13010111)
Supplement: Supplementary file 1 [file nutrients-13-00111-s001.pdf]

Table S1. Characteristics of included studies.

Author, Year, Country	Study design	Duration	Study population	Intervention	Control	Co-intervention	Compliance / Drop-out
Ghaderi, 2019, Iran [27]	Randomized, double-blind, placebo-controlled trial	12 weeks	*n* = 60, aged 25–65, 93.33% men, diagnosed with schizophrenia using DSM-IV-TR criteria with disease duration ≥2 years, PANSS score ≥55, treated with chlorpromazine (300–1000 mg/day, except clozapine) and anticholinergic agents (Trihexyphenidyl, 4–8 mg/day) during the last 6 months	Vitamin D3 and probiotic supplement:<br>- Vitamin D3: 50,000 IU every 2 weeks; DDE= 3,571.4 IU<br>- Probiotics: 8×10^9 CFU/day containing *Lactobacillus acidophilus, Bifidobacterium bifidum, Lactobacillus reuteri*, and *Lactobacillus fermentum* (each 2×10^9)	Placebo similar shape and packaging	None	Compliance: >90%<br>Drop out:<br>I: 13.33%<br>C: 13.33%<br>(Intention-to-treat analysis)
Jamilian, 2018, Iran [29]	Randomized, double-blind, placebo-controlled clinical trial	6 weeks	*n* = 87, women with GDM diagnosed by a "one-step" 2-h 75-g oral glucose tolerance test based on the ADA guidelines	Vitamin D and probiotic supplement:<br>- Vitamin D: 50,000 IU every 2 weeks; DDE= 3,571.4 IU<br>- Probiotics: 8×10^9 CFU/g probiotic containing *Lactobacillus acidophilus, Bifidobacterium bifidum, L. reuteri*, and *Lactobacillus fermentum* (each 2×10^9 CFU/g)	C1: 8×10^9 CFU/day of probiotic supplements<br><br>C2: Placebo<br><br>Similar in appearance, color, shape, size, smell and taste and packaging	Vitamin D3: 1000 IU and Vitamin B9: 400 mg, daily from the beginning of pregnancy, and Ferrous sulfate: 60 mg, daily from the second	Compliance: 100%<br>Drop out:<br>I: 0%<br>C1: 6.66%<br>C2: 10%

						trimester	
Ostadmohammadi, 2019, Iran [28]	Randomized, double-blind, placebo-controlled clinical trial	12 weeks	*n* = 60, aged 18–40 years, women with PCOS, diagnosed based on the Rotterdam criteria with BMI: 17–34 kg/m2 and insulin resistance: 1.4–4	Vitamin D and probiotic supplement: - Vitamin D: 50,000 IU every 2 weeks; DDE= 3,571.4 IU - Probiotics: 8×10^9 CFU/day containing *Lactobacillus acidophilus, Bifidobacterium bifidum, Lactobacillus reuteri* and *Lactobacillus fermentum* (each 2×10^9 CFU/g)	Placebo similar in appearance, color, shape, size, smell and taste and packaging	None	Compliance 100%; No drop out
Raygan, 2018, Iran [30]	Randomized, double-blind, placebo-controlled clinical trial	12 weeks	*n* = 60, age 45–85 years, 50% men, with T2DM diagnosed based on the criteria of the ADA and with CHD diagnosed as per the AHA with 2- and 3-vessel CHD	Vitamin D3 and probiotic supplement: - Vitamin D3: 50,000 IU every 2 weeks; DDE= 3,571.4 IU - Probiotics: 8×10^9 CFU/g containing *Lactobacillus acidophilus, Bifidobacterium bifidum, Lactobacillus reuteri, and Lactobacillus fermentum* (each 2×10^9 CFU/g)	Placebo similar in appearance, color, shape, size, smell and taste and packaging	None	Compliance >90% Drop out: I: 13.33% C: 13.33% (Intention-to-treat analysis)
Jafarnejad, 2017, Iran [31]	Randomized, double-blind, placebo-controlled clinical trial	6 weeks	*n* = 50, age 50–72 years, women with mild bone loss (osteopenia) diagnosed based on the World Health Organization criteria (T-score between −1.0 and −2.5)	Probiotic supplement: *Lactobacillus casei* 1.3×10^{10} CFU, *Bifidobacterium longum* 5×10^{10} CFU, *Lactobacillus acidophilus* 1.5×10^{10} CFU, *Lactobacillus*	Placebo similar in shape, size, odor, color and packaging	Vitamin D (200 IU daily) and Calcium (500 mg daily)	Compliance 100% Drop out: I: 20% C: 16%

				rhamnosus 3.5 × 10^9 CFU, *Lactobacillus bulgaricus* 2.5 × 10^8 CFU, *Bifidobacterium breve* 1 × 10^{10} CFU, and *Streptococcus thermophilus* 1.5 × 10^8 CFU/500 mg		
Savino, 2015, Italy [25]	Single-blind, randomized controlled, parallel-group trial	12 weeks	*n* = 105, newborns aged less than 10 days of life, 48.5% boys, with gestational age between 37 and 42 weeks, birth weight from 2,500 to 4,300 g, and normal physical examination	Vitamin D and probiotic supplement:<br>- Vitamin D3: 400 IU daily<br>- Probiotics: *Lactobacillus reuteri* DSM 17938 (10^8 CFU)	Vitamin D (400 IU daily)	None	No infants lost to follow-ups
Tazzyman, 2015, United Kingdom [26]	Double-blind, randomized, three-arm parallel design trial	12 weeks	*N* = 51, 7.8% men, with previous clinical diagnosis of IBS and met the Rome III criteria and stratified according to vitamin D status at baseline (deficient: 25(OH)D < 20 ng/mL; repleted: 25(OH)D > 20 ng/mL)	Vitamin D3 and probiotic supplement:<br>-Vitamin D3: sublingual liquid spray, 3000 IU daily<br>- Probiotics: *Lactobacillus acidophilus*, CUL60 (NCIMB 30157), CUL21 (NCIMB 30156), *Bifidobacterium bifidum* CUL20 (NCIMB 30153) and *Bifidobacterium animalis subsp. lactis* CUL34 (NCIMB 30172) 2.5 × 10^{10} CFU per capsule	C1: Double placebo<br><br>C2: Placebo and Vitamin D3 (400 IU daily)<br><br>Similar in form, containing identical buffers	None	Compliance: 98%<br>Drop out: 0%

25(OH)D: 25-hydroxyvitamin D; ADA: American Diabetes Association; AHA: American Heart Association; BMI: Body Mass Index; C: Control; CFU: Colony Forming Units; CHD: Coronary Heart Disease; DDE: Daily Dose Equivalent; DSM-IV-TR: Diagnostic and Statistical Manual of Mental Disorders, Fourth Edition,

Text Revision; GDM: Gestational Diabetes Mellitus; I: Intervention; IBS: Irritable Bowel Syndrome; IU: International Unit; PANSS: The Positive and Negative Syndrome Scale; PCOS: Polycystic Ovary Syndrome; T2DM: Type 2 Diabetes Mellitus; TDD: Total Daily Dose.

Table S2. Outcomes and results of included studies.

Author, Year, Country	Outcome measures	Results	Conclusion
Ghaderi, 2019, Iran [27]	BMI: weight in kg divided by height in meters squared (height and weight measured without shoes and in light clothing by a trained staff)<br>Serum 25-hydroxyvitamin D: ELISA kit<br>Severity of psychiatric symptoms: PANSS<br>Domains of cognitive function: BPRS scores<br>TAC: method of ferric reduction antioxidant power developed by Benzie and Strain<br>GSH: Beutler method<br>MDA: Thiobarbituric acid reactive substances spectrophotometric Test<br>Serum hs-CRP: ELISA kit<br>NO: Griess Method<br>Serum insulin: ELISA kit<br>HOMA-IR and QUICKI: calculated using standard formula<br>FPG and lipid profiles: Enzymatic kits	At baseline and endline: No significant difference between-groups, in height, age, weight, BMI and METs<br><br>At baseline: Significant difference between-groups for positive PANSS score, BPRS, GSH and plasma NO<br><br>At endline:<br>In the I group compared with the C group:<br>Significant greater decrease in MDA (−0.3 ± 0.9 vs. +0.2 ± 0.4 μmol/L), serum hs-CRP (−2.3 ± 3.0 vs. −0.3 ± 0.8 mg/L), FPG (−7.0 ± 9.9 vs. −0.2 ± 9.9 mg/dL), serum insulin (−2.7 ± 2.3 vs. +0.4 ± 2.0 μIU/mL), HOMA-IR (−0.8 ± 0.7 vs. + 0.1 ± 0.7), TG (−7.8 ± 25.2 vs. +10.1 ± 30.8 mg/dL), TC (−4.9 ± 15.0 vs. +5.9 ± 19.5 mg/dL), and TC/HDL-C (−0.1 ±0.6 vs. +0.3 ± 0.8)<br>Significant greater increase in 25-hydroxyvitamin D (+ 9.1 ± 4.1 vs. +0.2 ± 0.4 ng/mL), general PANSS score (−3.1 ± 4.7 vs. +0.3 ± 3.9), total PANSS score (−7.4 ± 8.7 vs. −1.9 ± 7.5), plasma TAC (+51.1 ± 129.7 vs. −20.7 ± 53.3 mmol/L), QUICKI (+0.02 ± 0.01 vs. +0.0003 ± 0.01)	Probiotic and vitamin D co-supplementation for 12 weeks to patients with chronic schizophrenia had beneficial effects on the general and total PANSS scores, as well as their metabolic profiles, compared with placebo

		No significant difference in the change of BPRS score and other metabolic profiles<br><br>In the analysis adjusting for baseline values of biochemical parameters, age and BMI, and controlling for potential confounders:<br>The difference in changes in TC/HDL between the two groups became non-significant<br>The difference in changes in negative PANSS score, BPRS and plasma GSH became statistically significant<br>Other metabolic profiles did not change statically	
Jamilian, 2018, Iran [29]	BMI: weight in kg divided by height in meters squared (height and weight measured without<br>shoes and in light clothing by a trained staff)<br>Polyhydramnios: sonographic estimation method at post-intervention and defined as an AFI in excess of 25 cm<br>Preterm delivery: defined as delivery occurred at <37 weeks of pregnancy<br>Newborn's macrosomia: defined as birth weight of >4000 g. 2.5<br>Serum 25-hydroxyvitamin D: ELISA kit<br>Serum insulin: ELISA kit<br>HOMA-IR and QUICKI: calculated according to the standard formula<br>FPG, serum TG, VLDL-C, TC, LDL-C and HDL-C: enzymatic kits<br>Serum hs-CRP: ELISA kit	At baseline and endline: No significant difference between-groups, in age, height, weight, BMI, METs and intakes of macro- and micronutrients<br><br>At endline:<br>In the I group compared with the C1 group<br>Significant greater decrease in TG (β −15.82 mg/dL), VLDL-C (β −3.16 mg/dL) and hs-CRP (β −0.32 mg/L)<br>Significant greater increase in serum 25-hydroxyvitamin D (β 16.16 ng/mL), TAC (β 63.26 mmol/L) and GSH (β 53.61 mmol/L)<br>Lower incidence of hyperbilirubinemiain newborns (10.0% vs. 13.8%)<br>Lower incidence of newborns' hospitalization (10.0% vs. 10.3%)<br>No significant changes in other pregnancy outcomes<br><br>In the I group compared with C2 group:<br>Significant greater decrease in FPG (β −10.99 mg/dL), serum insulin (β −1.95 mIU/mL), HOMA-IR (β −0.76; 95%), TG (β −37.56 mg/dL), VLDL-C (β	High dose of vitamin D and probiotic co-supplementation for 6 weeks to women with GDM had beneficial effects on metabolic status and newborns' outcomes compared with placebo and low dose of vitamin D or probiotic supplementation and a low dose of vitamin D

	Plasma NO: Griess method<br>TAC: method of ferric reducing antioxidant power developed by Benzie and Strain<br>GSH: Beutler method<br>MDA: Thiobarbituric acid reactive substances spectrophotometric Test<br>Newborns' hyperbilirubinemia: when the total serum bilirubin levels were at ≥15 mg/dL (257 mmol/L) among infants 25–48 h old, 18 mg/dL (308 mmol/L) in infants 49–72 h old, and 20 mg/dL (342 mmol/L) in infants >72 h old	−7.51 mg/dL), HDL/TC B: −0.52), hs-CRP (β −1.80 mg/L) and MDA (β −0.43 mmol/L)<br>Significant greater increase in 25-hydroxyvitamin D (β 18.21 ng/mL), QUICKI (β 0.01) HDL-C (β 4.09 mg/dL) and TAC (β 97.77 mmol/L)<br>No significant changes in other metabolic parameters<br>Lower incidence of hyperbilirubinemia in newborns (10.0% vs. 35.7%)<br>Lower incidence of newborns' hospitalization (10.0% vs. 32.1%)<br>No significant changes in other pregnancy outcomes<br><br>In the C1 group compared with the C2 group<br>Significant greater decrease in FPG (β −8.60 mg/dL), Insulin (β −1.34 μIU/mL), HOMA-IR (β −0.54), TG (β −21.73 mg/dL), VLDL-C (β −4.34 mg/dL) and hs-CRP (β −1.36 mg/L), and MDA (β −0.50 μmol/L)<br>Significant greater increase in serum 25-hydroxyvitamin D (β 2.05 ng/mL)	
Ostadmohammadi, 2019, Iran [28]	Hirsutism: mFG scoring system<br>Mental health: BDI, GHQ−28 and DASS<br>Quality of sleep: PSQI<br>Serum 25-hydroxyvitamin D: ELISA kit<br>Serum total testosterone and SHBG: ELISA kits<br>hs-CRP: ELISA kit<br>Plasma NO: Griess method<br>TAC: Benzie and Strain method<br>GSH: Beutler method	At baseline:<br>No significant difference between groups for mean age, height and dietary macro- and micro-nutrient intakes.<br><br>At endline:<br>In the I group compared with the C group:<br>Significant greater decrease in BDI (β −0.58), GHQ (β − 0.93), DASS (β − 0.90), total testosterone (β − 0.19 ng/mL), hirsutism (β − 0.95), hs-CRP (β − 0.67 mg/L) and MDA (β − 0.25 μmol/L)<br>Significant greater increase in TAC (β 82.81 mmol/L) and GSH (β 40.42 μmol/L)	Vitamin D and probiotic co-supplementation for 12 weeks to women with PCOS had beneficial effects on mental health parameters, but did not affect serum SHBG, plasma NO levels, acne, alopecia and PSQI, compared with placebo

	MDA: Thiobarbituric acid reactive substances spectrophotometric Test	No significant effect on serum SHBG and plasma NO levels, acne, alopecia and PSQI	
Raygan, 2018, Iran [30]	Serum 25-hydroxyvitamin D: ELISA<br>FPG and lipid profiles: Enzymatic kit<br>Insulin: ELISA kit<br>HOMA-IR and QUICKI: standard formula<br>Hs-CRP: ELISA kit<br>Plasma TAC: Benzie and Strain method<br>GSH: Beutler and Gelbart method<br>MDA: spectrophotometric test<br>NO: Griess method<br>SBP and DBP: sphygmomanometer (Not detailed)<br>Mental health: BDI, BAI, GHQ–28	At baseline and endline:<br>No significant differences between-groups in mean age, height, weight, BMI and METs and macro and micronutrient intakes<br><br>At endline:<br>In the I group compared with the C group:<br>Significant greater decrease in BDI (−2.8 ± 3.8 vs. −0.9 ± 2.1), BAI (−2.1 ± 2.3 vs. −0.8 ± 1.4) and GHQ scores (−3.9 ± 4.1 vs. −1.1 ± 3.4), Insulin (µIU/mL) (−2.8 ± 3.8 vs. +0.2 ± 4.9), HOMA-IR (−1.0 ± 1.6 vs. −0.1 ± 1.5), and hs-CRP (ng/mL) (−950.0 ± 1811.2 vs. +260.5 ± 2298.2)<br><br>Significant greater increase in 25-hydroxyvitamin D (ng/mL) (+11.8 ± 5.9 vs. +0.1 ± 1.4), QUICKI (+0.03 ± 0.04 vs. −0.001 ± 0.01), serum HDL-cholesterol (mg/dL) (+2.3 ± 3.5 vs. −0.5 ± 3.8), plasma NO (µmol/L) (+1.7 ± 4.0 vs. −1.4 ± 6.7) and plasma TAC (mmol/L) (+12.6 ± 41.6 vs. −116.9 ± 324.2)<br>No significant different changes in FPG, Triglycerides, VLDL-Cholesterol, LDL-Cholesterol, GSH, MDA, SBP and DBP	Vitamin D and probiotic co-supplementation for 12 weeks to diabetic people with CHD had beneficial effects on mental health, glycemic control, HDL-cholesterol levels, hs-CRP, NO and TAC, but did not affect other metabolic profiles and blood pressures, compared with placebo
Jafarnejad, 2017, Iran [31]	Nutrient intake: 3-day dietary recall (2 weekdays and one weekend day), through monthly interview throughout the study period; nutrient analysis: by Nutritionist IV software modified for Iranian foods<br>Physical activity: daily physical activity questionnaires validated by	At baseline: No significant differences between-groups<br><br>At endline:<br>Significant between-group differences in BALP (U/L) (I: 19.65 ± 1.66 at baseline and 16.53 ± 0.90 at endline vs. C: 17.81 ± 1.35 at baseline and 18.63 ± 1.29 at endline); CTX (ng/ml) (I: 0.41 ± 0.02 at	Supplementation with probiotics, vitamin D and calcium for 6 weeks to postmenopausal osteopenic women showed a possible role in suppressing bone resorption and bone

	Kelishady et al. and calculated as metabolic equivalents/day<br>Body weight: measured wearing light clothes without shoes using digital scales with 100-g precision<br>Height: measured using a stadiometer with 0.5-cm precision in a normal standing position without shoes.<br>BMI: weight in kilograms divided by height in meters squared<br>BMD: dual energy X-ray absorptiometry<br>Bone and pro-inflammatory biomarkers (TNF-α and IL-1b), Total serum levels of BALP, Osteocalcin, CTX, Vitamin D, RANKL, Osteoprotegrin, Serum TNF-α and IL-1b, Serum PTH, Urinary deoxypyridinoline: ELISA kits<br>Serum calcium, phosphorus, magnesium, albumin, creatinine, alkaline phosphatase, and urinary amounts of calcium, phosphorus, magnesium, and creatinine: Pars Azmoon kits	baseline and 0.35 ± 0.02 at endline vs. C: 0.45 ± 0.02 at baseline and 0.42 ± 0.02 at endline); TNF-α (pg/ml) (I: 4.24 ± 0.5 at baseline and 3.73 ± 0.43 at endline vs. 3.83 ± 0.47 at baseline and 4.32 ± 0.5 at endline); PTH (pg/ml) (I: 31.92 ± 1.39 at baseline and 29.05 ± 1.53 at endline vs. C: 30.65 ± 1.44 at baseline and 32.81 ± 1.72 at endline)<br>No significant between-group difference in Spinal BMD, Total hip BMD, RANKL, osteoprotegrin, RANKL/ osteoprotegrin ratio, deoxypyridinoline, osteocalcin, IL-1, Vitamin D, serum calcium, 24-Hour urinary Calcium, Serum phosphorus, 24-Hour urinary phosphorus, Serum magnesium, 24-Hour urinary magnesium, Serum creatinine, 24-Hour urinary creatinine, ALP, Albumin	turnover, but did not affect bone density and other serum indicators compared with placebo, vitamin D and calcium
Savino, 2015, Italy [25]	Administration of pain-relieving agents (cimetropium bromide at least three<br>times per week or simethicone at least five times per<br>week): daily reporting by parents	In the I group compared with the C group:<br>- Significantly lower use of pain-relieving agents: Cimetropium bromide: RR: 0.04 (95%CI: 0.01–0.31); Simethicone: RR: 0.24 (95%CI: 0.14–0.41)<br>- Significantly lower use of infant formula: RR: 0.37 (95%CI: 0.17- 0.80)<br>- Significantly lower number of calls to the pediatrician: 5.04 ± 2.64 vs. 8.40 ± 3.58	Vitamin D and probiotic co-supplementation for 12 weeks to newborns was associated with a reduction of pediatric consultations for infantile colic, use of pain-relieving agents and of

	% of infants switching from exclusive breastfeeding to partial or exclusive formula feeding: not detailed<br>Number of phone-calls and visits due to infantile colic: noted by the pediatrician.	- Significantly lower number of visits in the pediatric ambulatory: 2.66 ± 1.77 vs. 4.98 ± 1.89	infant formula, compared with vitamin D supplementation
Tazzyman, 2015, UK [26]	Serum 25(OH)D: Cobas e411 automated immunoassay<br>Dietary intake: Food frequency questionnaire analyzed using FETA open source software<br>IBS symptom: questionnaire assessing abdominal pain (pain severity and number of days with pain), bloating, bowel habits (minimum and maximum bowel movement per day and satisfaction with bowel habit) and quality of life	At baseline: No significant differences between-groups<br><br>At endline:<br>In the I and C2 groups compared with the C1 group:<br>- Significantly higher 25OHD (ng/mL) (37.2 ±9.3 and 37.1 ± 11.7 vs. 25.3 ± 8.0)<br>No significant between-group differences for any symptom tested, and total symptom severity (same results obtained for participants who were 25(OH)D-deficient at baseline)	Vitamin D and probiotic co-supplementation had no significant effect on the symptoms of IBS, compared with vitamin D alone, or placebo

25(OH)D: 25-hydroxyvitamin D; AFI: Amniotic Fluid Index; BAI: Beck Anxiety Inventory; BALP: Bone-Specific Alkaline Phosphatase; BDI: Beck Depression Inventory; BMD: Bone Mineral Density; BMI: Body Mass Index; BPRS: Brief Psychiatric Rating Scale; C: Control; CHD: Coronary Heart Disease; CI: Confidence Interval; CXT: Collagen Type 1 Cross-Linked C-Telopeptide; DASS: Depression Anxiety and Stress Scale; DBP: Diastolic Blood Pressure; ELISA: Enzyme-Linked Immunosorbent Assay; FBG: Fasting plasma glucose; GDM: Gestational Diabetes Mellitus; GHQ-28: General Health Questionnaire-28; GSH: Total Glutathione; HDL-C: High-Density Lipoprotein Cholesterol; HOMA-IR: Homeostasis Model of Assessment-Insulin Resistance; hs-CRP: High-Sensitivity C-Reactive Protein; I: Intervention; IBS: Irritable Bowel Syndrome; IL: Interleukin; LDL-C: Low-Density Lipoprotein Cholesterol; MDA: Malondialdehyde; MET: Metabolic Equivalent; mFG: modified Ferriman-Gallwey; NO: Nitric oxide; PCOS: Polycystic Ovary Syndrome; PSQI: Pittsburgh Sleep Quality Index; PTH; Parathyroid Hormone; QUICKI: Quantitative Insulin Sensitivity Check Index; RANKL: Serum Total Receptor Activator of Nuclear Factor-kB Ligand; RR: Relative Risk; SBP: Systolic Blood Pressure; SHGB: Sex Hormone-Binding Globulin; T2DM: Type 2 Diabetes Mellitus ; TAC: Total Antioxidant Capacity ; TC: Total cholesterol; TG: TNF: Tumor Necrosis Factor; Triglycerides; VLDL-C: Very Low-Density Lipoprotein Cholesterol. [1]Significance obtained for the time × group interaction, computed by analysis of the one-way repeated measures ANOVA. [2]Outcome measures refer to the change in values of measures of interest between baseline and endline in each group. [3]β: difference in the mean outcomes measures between treatment groups, and significance obtained from multiple regression model (adjusted for baseline values of each variable).
